# Varus distal femoral osteotomy in young adults with valgus knee

**DOI:** 10.1186/1749-799X-4-15

**Published:** 2009-05-13

**Authors:** Farzad Omidi-Kashani, Ibrahim G Hasankhani, Mahdi Mazlumi, Mohamad H Ebrahimzadeh

**Affiliations:** 1 Department of orthopedic surgery, Qhaem hospital, Mashhad University of Medical Sciences, Mashhad, Iran; 2 Department of orthopedic surgery, Imam Reza hospital, Mashhad University of Medical Sciences, Mashhad, Iran

## Abstract

**Background:**

Musculoskeletal disorders specially knee osteoarthritis are the most common causes of morbidity in old patients. Disturbance of the mechanical axis of the lower extremity is one of the most important causes in progression of knee osteoarthritis. The purpose of the present study was to analyze the surgical results of distal femoral varus osteotomy in patients with genu valgum.

**Methods:**

In this study, after recording history and physical examination, appropriate radiographs were taken. We did varus distal femoral osteotomy by standard medial subvastus approach and 90-angle blade plate fixation then followed the patients clinically and radiographically.

**Results:**

This study was done on 23 knees (16 patients) age 23.3 years (range, 17 to 41 years). The mean duration of following up was 16.3 months (range, 8 to 25 months). Based on paired T test, there were statistically significant difference between pre- and postoperative tibiofemoral and congruence angles (p < 0.001, t = 21.3 and p < 0.001, t = 10.1 respectively). Pearson correlation between the amount of tibiofemoral and congruence angle correction was also statistically significant (p = 0.02 and r = 0.46).

**Conclusion:**

Distal femoral varus osteotomy with blade plate fixation can be a reliable procedure for the treatment of valgus knee deformity. In this procedure, with more tibiofemoral angle correction, more congruence angle correction can be achieved. Therefore, along with genu valgum correction, the patella should be stabilized simultaneously.

## Background

Musculoskeletal disorders specially knee osteoarthritis are the most common causes of morbidity in old patients [1]. Disturbance of the mechanical axis of the lower extremity is one of the most important causes in progression of knee osteoarthritis [2,3].

Whereas high tibial osteotomy has been used successfully to treat medial compartment disease with varus deformity, the results of tibial osteotomy for valgus deformity have varied [4-6]. Because of the inherent valgus femorotibial angulation at the knee joint, tibial varus osteotomy could be used to correct a femorotibial angulation of no more than 12° of valgus. Correction of a larger deformity creates a varus or medial tilt of the joint line which is subjected to increased lateral shear forces, and this tends to cause the femur to subluxate medially on the tibia during gait [7-9].

In light of this, several authors stated that if a knee shows an anatomic tibiofemoral angle >10–12° of valgus or if the plane of the joint deviates from the horizontal in the superolateral direction more than 10°, a distal femoral varus osteotomy is the preferred method of limb realignment [8,10-12]. This procedure corrects deformity in the lower femur, which is more pronounced than in knees with varus deformity. It also restores the orientation of the joint line toward the horizontal and does not disturb medial collateral ligament stability [13-15].

The purpose of the present study was to study and analyze the surgical results of distal femoral varus osteotomy in patients with genu valgum.

## Methods

Between June 2000 and November 2006, 25 distal femoral osteotomies were performed in 18 patients (14 women and 4 men) at our institution.

Standing anteroposterior and lateral radiographs as well as Merchant radiographs of the knee were made preoperatively and at the time of each follow-up. The sulcus and congruence angles of the patellofemoral joint were measured with the use of the method described by Merchant et al. before the osteotomy and at the time of the latest follow-up [16]. Subluxation of the patella was defined as being present if the congruence angle was >16° on the Merchant radiograph [17].

**Figure 1:**
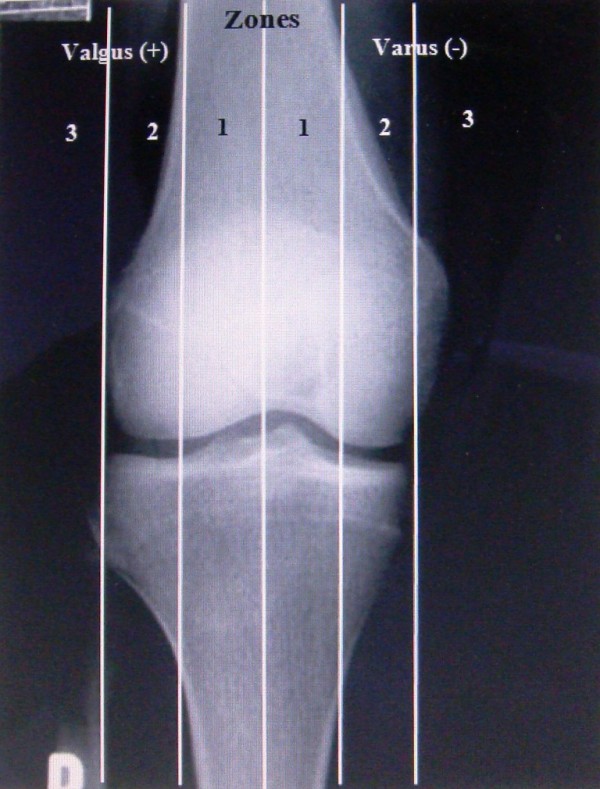
**The relation between the mechanical axis and the knee**.

We used the method described by Stevens PM et al. who believed that the knee can be divided into four radiographic quadrants (figure 1), designating varus as negative and valgus as positive [18]. The mechanical axis measured on a full-length film can be readily correlated to any of these zones with little interobserver error. Plus or minus zone I, the central quadrants, represent physiologic deformities. Plus or minus zone II often correlate with symptomatic deformities that may warrant surgical intervention. Plus or minus zone III are outside the confines of the knee and usually warrant surgical intervention.

Our surgical indications were genu valgum with the mechanical axis in plus zone III, the intermalleolar distance >5 cm, a painful deformity associated with a valgus tibiofemoral angulation of > 12° and narrowing of the lateral joint space, patellar instability, circumduction gait, and cosmetic concerns. We tried to mainly consider the deformity with a deviated mechanical axis as a principle indication for surgery, even though the patient is completely asymptomatic. Without intervention, biomechanically the knee most likely has an increased risk of developing early osteoarthritis [19,20].

Our exclusion criteria included severe arthritis of the medial compartment of the knee, severe tricompartment osteoarthritis, and tibiofemoral subluxation greater than one centimeter.

The Hospital for Special Surgery knee-rating system was used for the clinical evaluation of all patients [21]. Laxity of the medial collateral ligament was classified according to Scuderi and Scott classification [22]. All patients were designed to follow regularly at 6 weeks, 3 months, 6 months, 1 year, and then yearly thereafter. Two patients were lost to follow-up and so excluded them from the study.

### Surgical Technique

The technique for distal femoral varus osteotomy was based on the method described by McDermott et al [13]. The osteotomy was performed through a medial incision with removal of a 5 to 10-mm medially based wedge of bone from the distal part of the femur. A prebent 90° dynamic-compression blade plate was inserted into the femoral condyle, parallel to the knee-joint line. The osteotomy site was closed with the plate in contact with the medial femoral cortex. This spontaneously achieved a tibiofemoral angle of approximately 0° and then final osteosynthesis was performed with the dynamic-compression plate. A cortical or cancellous bone lag screw was inserted through the hole above the bend in the blade-plate, across the osteotomy site, to provide additional stability. A lateral retinacular release was performed in 6 knees because of lateral subluxation of the patella [23] or excessive lateral retinacular tightness palpated intraoperatively and after fixation of the osteotomy site.

Postoperatively, the affected limb was immobilized in a hinged knee brace until healing of the osteotomy site occurred. Patients began to use a continuous-passive-motion machine 48 hours after the operation and continued to use it until 90° of knee flexion was achieved. Non-weight bearing walking was commenced on the second postoperative day and was continued until initial healing of the osteotomy site had been confirmed radiographically, usually after six weeks of follow-up. Full weight-bearing was only permitted after 3 months of follow-up and after radiographs showed good healing of the osteotomy site.

### Statistical Analysis

The preoperative and most recent knee score were compared with use of the paired T test. The level of significance was set at p < 0.05. All analyses were performed with use of Statistical Package Science Software (SPSS version 10).

## Results

We could successfully follow 23 distal femoral osteotomies in 16 patients (12 women and 4 men) at our institution. All of the seven bilaterally involved patients were women. The mean age of the patients at the time of the index operation was 23.3 years (range, 17 to 41 years). Of these, 6 knees had patellar subluxation. No patient with complete patellar dislocation was present in our patients.

The mean knee score improved from 90.7 points (range, 77 to 96 points) preoperatively to 98.13 points (range, 93 to100 points) at the time of the most recent follow-up (p > 0.05).

The laxity of the medial collateral ligament was classified as grade 1 for 8 knees, and grade 2 for 5; the remaining 10 knees had no laxity. Knee stability was achieved in all patients after solid union of the osteotomy site. The mean pre- and postoperative tibiofemoral and congruence angles were shown in table 1.

**Table 1:** Mean pre- and postoperative tibiofemoral and congruence angle

**Angle** **(Degree)**	**Preoperative** **Mean (SD^❈^)**	**Postoperative** **Mean (SD)**	**Difference** **Mean (SD)**
**Tibiofemoral**	20.3 (4.2)	0.13 (2.9)	20.2 (4.5)
**Congruence**	14.8 (7.2)	1.48 (3.8)	13.3 (6.2)

^❈^SD: Standard Deviation

Before surgery, in 43.5% of knees, the mechanical axis passed from zone II, and in other 56.5%, zone III was the site of the drawn mechanical axis. Postoperatively, all of the knees' mechanical axes were laid in zone I.

The mean duration of follow-up was 16.3 Months (range, 8 to 25 months). Union of the osteotomy site was achieved in all but one knee, with an average time to union of 4.1 months (range, 2 to 6 months). An autogenous iliac crest bone graft was required to achieve healing of one nonunion. Superficial wound infection occurred in 2 (8.7%) knees; treated successfully with oral antibiotics. Implant failure occurred in 1(4.3%) patient aged 23 years who had fallen 2 months after surgery and bent the plate, then suffered nonunion of the osteotomy. Revision of the fixation using a blade plate, supplemented with bone grafting, resulted in satisfactory union.

Clinical evaluation revealed no loss of knee motion compared with the preoperative examination. The distance between the medial malleoli was reduced in all subjects; most of the patients could approximate ankles and knees simultaneously, indicating complete correction of genu valgum. Circumduction gait, which was common before surgery, was corrected in all patients. No patient had varus laxity >5 mm. There were no patients with limb-length inequality >1.5 cm.

## Discussion

Although the mean knee score in our patients improved with surgery, in contrast to most other studies [10,11,14,15], this difference was not statistically significant mostly because the operation was done in younger healthy patients (not in osteoarthritic patients). The main aim of our operation was to avoid late knee osteoarthritic derangement.

In our study, based on paired T test, there were statistically significant difference between pre- and postoperative tibiofemoral and congruence angles (p < 0.001, t = 21.3 and p < 0.001, t = 10.1 respectively). Therefore, the procedure could sufficiently correct the deformity.

**Figure 2:**
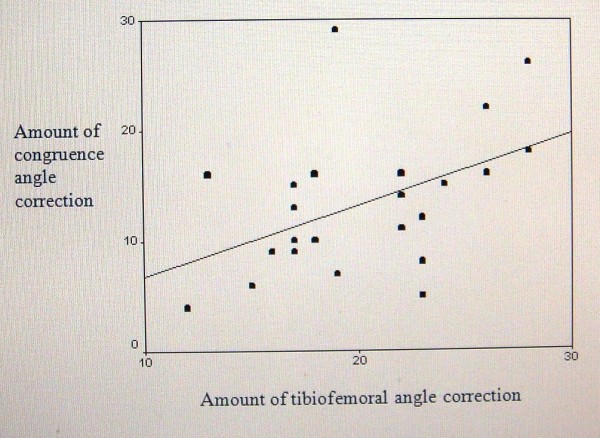
**Correlation between tibiofemoral and congruence angle correction**.

Pearson correlation between the amount of tibiofemoral and congruence angle correction was statistically significant (p = 0.02 and r = 0.46). In respect to positive "r", one can concluded that with more tibiofemoral angle correction, more congruence angle correction can be achieved. In other word, along with genu valgum correction, the patella should be stabilized simultaneously (figure 2).

The method of fixation after the osteotomy appears to have a great influence on the results of this procedure. Use of blade-plate for fixation at the osteotomy site has been associated with a high healing rate and promising results in short-term follow-up studies [10,11,13]. In a study was done by Wang JW et al. on 30 knees that distal femoral varus osteotomy was fixed with a 90° blade-plate, reported that 83% had satisfactory result and only one nonunion occurred [11].

Healy WL et al. evaluated 23 distal femoral varus osteotomies at an average of 4 years postoperatively. The average tibiofemoral angle preoperatively was 18° of valgus, which was corrected to an average of 2° of valgus. According to the Hospital for Special Surgery Knee score, 19 (83%) of the 23 knees were rated as good or excellent. 15 osteotomies were performed on osteoarthritic knees and all but one (93%) knee were rated as good or excellent [14].

Aglietti P et al reported the results of 18 distal femoral varus osteotomies in patients with osteoarthritis of the lateral compartment of the valgus knee. The osteotomy site was fixed with a 90° blade-plate. With an average follow-up of 9 years, they cited 77% good or excellent results according to the Knee Society rating system. No patients had nonunion or infection. They advised the procedure for the treatment of symptomatic valgus knee in both young and older active patients [10].

In another study conducted by Mathews J and coauthors, they described 21 patients treated with distal femoral varus osteotomies immobilized by casting, staples and casting, and rigid internal fixation with an AO blade plate. They reported satisfactory results only in those patients who had less severe degrees of osteoarthritis confined to the lateral compartment, adequate correction of valgus deformity (the anatomic axis within 2° from zero), and rigid internal fixation to permit postoperative early mobilization [15].

## Conclusion

In conclusion, although a prospective trial is required to investigate the optimal postoperative alignment angle, distal femoral varus osteotomy with blade plate fixation can be a reliable procedure for the treatment of valgus knee deformity. In this procedure, with more tibiofemoral angle correction, more congruence angle correction can be achieved. Therefore, along with genu valgum correction, the patella should be stabilized simultaneously

## Competing interests

The authors declare that they have no competing interests.

## Authors' contributions

FOK, the senior surgeon and has made substantial contributions to conception and design of the manuscript. IGH has been involved in drafting the manuscript, participated in the sequence alignment. MM has made substantial contributions to acquisition of data from literature. MHE has had substantial role in preparing and revising the manuscript.
